# Nomogram analysis and external validation to predict the risk of lymph node metastasis in gastric cancer

**DOI:** 10.18632/oncotarget.14535

**Published:** 2017-01-06

**Authors:** Shi Chen, Run-Cong Nie, Li-Ying OuYang, Yuan-Fang Li, Jun Xiang, Zhi-Wei Zhou, YingBo Chen, Jun-Sheng Peng

**Affiliations:** ^1^ The 6th Affiliated Hospital, Sun Yat-Sen University, YuanCun ErHeng Road, TianHe District, 510655, Guangzhou, China; ^2^ Department of Gastropancreatic Surgery, Sun Yat-Sen University Cancer Center, 510060, Guangzhou, China; ^3^ Department of Intensive Care Unit, Sun Yat-Sen University Cancer Center, 510060, Guangzhou, China

**Keywords:** gastric cancer, lymph node metastasis, risk, nomogram

## Abstract

**Aim:**

To identify risk factors for lymph node metastasis using a nomogram for gastric cancer patients to predict lymph node metastasis.

**Results:**

The Chi-square test and the logistic regression showed that the Boarrmann type, preoperative CA199 level, T stage and N stage by CT scan were independent risk factors. The concordance index (C-index) was 0.786 in the internal validation of the Nomogram model. In the external validation, the C-index was 0.809, and the AUC was 0.894. The total accuracy of the prediction was 82.2%, and the false-negative rate was 5.4% with a cut-off value set at 0.109.

**Materials and Methods:**

The study consisted of 451 patients with a histological diagnosis of gastric cancer with 0 or 1 lymph node metastasis from the Sun Yat-sen University Cancer Center as the development set, and the validation set consisted of 186 gastric cancer patients from the Sixth Affiliated Hospital of Sun Yat-Sen University. A Chi-square test and a logistic regression analysis were used to compare the clinicopathological variables and lymph node metastasis. The C-index and ROC curve were computed for comparisons of the nomogram's predictive ability.

**Conclusions:**

We developed and validated a nomogram to predict lymph node metastasis in gastric cancer before surgery. This nomogram can be broadly applied, even in general hospitals, and is useful for decisions regarding treatment programs for patients.

## INTRODUCTION

Although the morbidity of gastric cancer continues to decline in North America and Western Europe, it remains the second most malignant tumor in China [1, 2]. D2 gastrectomy has now become the standard surgery for gastric cancer, especially advanced gastric cancer [3, 4]. However, with the development of screening, an increasing number of early stage gastric cancer cases are found. Because D2 gastrectomy commonly causes more postoperative complication and mortality, surgeons believe that endoscopic mucosa resection (EMR) is the best fit the early stage gastric cancer. However, lymph node metastasis is the most common metastatic path for gastric cancer, even in early stage gastric cancers. The prevalence of lymph node metastasis in early gastric cancer (EGC) is reported to be in the range of 7.7 to 19.4% [5–7]. If EMR is undertaken as a radical surgery in these early stage gastric cancer patients, it is difficult to avoid recurrence. Thus, precise predictions of lymph node metastasis are very important for gastric cancer patients, especially at the early stage.

Sentinel lymph nodes were first used to predict lymph node metastasis, and it has been proven to be effective in malignant melanoma and breast cancer [8–10]. However, in gastric cancer, the drainage of the lymph node is net-style, which is more complicated than in melanoma and breast cancer. It is difficult for clinicians to accurately locate the sentinel lymph node, even with the development of nano lymph node tracers and ^99^mTc tin colloid [10, 11].

CT scan and ultrasound endoscopy are currently commonly used in the clinic to evaluate the preoperative staging of gastric cancer before surgery. The accuracy of these tools is still not satisfying, according to a report by Feng XY et al. The accuracy, regarding the presence of LM, was 61.1% in MSCT studies [12]. Indeed, it is very important to have a model to predict the risk of lymph node metastasis because an accurate prediction of lymph node metastasis is mandatory to reduce the extent of surgery without hampering the oncological safety of EGC patients.

Nomograms have been widely used for predicting the prognosis of malignant cancer patients and quantifying the risk factors of lymph node metastasis in breast cancer and prostate cancer [13–15]. The aim of the present study was to identify risk factors of lymph node metastasis using a nomogram for patients with gastric cancer to guide treatment management.

## RESULTS

### Correlations between lymph node metastasis and the clinicopathological features of gastric cancer patients with 0 or 1 lymph node metastasis in the SYSUCC group

Among the 451 gastric cancer patients, lymph node metastasis was associated with tumor size, Boarrmann type, histological type, preoperative CEA and CA199 level, T stage and N stage by CT scan (all *p* < 0.05). The results are shown in Table 1.

**Table 1:** Correlation between solitary lymph node metastasis and clinicopathological variables in the development set

Clinicopathological parameters	Development set (*n* = 451)					Validation set (*n* = 186)				
	n^a^	lymph node metastasis		χ2	*P* value	n^a^	lymph node metastasis		χ^2^	*P* value
		Negative	Solitary				Negative	Positive		
**All**	451	350	101			186	59	127		
**Tumor size (cm)**				25.579	< 0.001				21.038	< 0.001
< 5	288	248	44			96	45	51		
≥ 5	163	102	57			90	14	76		
**Boarrmann type**				52.902	< 0.001				78.276	< 0.001
I	44	40	4			29	27	2		
II	226	201	25			86	30	56		
III	177	107	70			61	2	59		
IV	4	2	2			10	0	10		
**Histological Grade**				7.859	0.049				13.228	0.004
high-differentiated	24	23	1			13	10	3		
median- differentiated	143	116	27			52	15	37		
low-differentiated	230	169	61			102	29	73		
undifferentiated	54	42	12			19	5	14		
**Tumor infiltration**				34.241	< 0.001				53.372	< 0.001
T1	121	112	9			28	25	3		
T2	83	70	13			18	6	12		
T3	14	7	7			99	23	76		
T4a	206	144	62			41	5	36		
T4b	27	17	10			0	0	0		
**Preoperative CEA**				7.086	0.008				2.356	0.167
Normal	389	310	79			131	46	85		
Elevated	62	40	22			55	13	42		
**Preoperative CA19-9**				11.484	0.001				7.598	0.006
Normal	351	281	70			148	54	94		
Elevated	30	16	14			38	5	33		
**N stage by CT scan**				32.811	< 0.001				34.510	< 0.001
Negative	332	280	52			72	41	31		
Positive	119					114	18	96		

^a^Numbers of cases in each group. * Statistically significant (*P* < 0.05).

### The nomogram for the prediction of metastatic lymph nodes

We used a logistic regression model for the multiple variable analysis of the lymph node metastasis for gastric cancer. We observed that the Boarrmann type, T stage evaluated by CT, N stage evaluated by the CT and the preoperative serum CA19-9 level were independent risk factors for lymph node metastasis in gastric cancer. The results are shown in Table 2.

**Table 2:** Multivariate analyses of the lymph node metastasis in the development set (Logistic regression model)

Variable	OR	95% CI	*P* value
Lymph node metastasis risk in gastric cancer patients			
**Tumor Size**	0.911	0.469–1.770	0.784
**Histological grade**			0.548
high-differentiated	Ref	Ref	
median- differentiated	0.530	0.052–5.440	
low-differentiated	0.691	0.232–2.053	
undifferentiated	1.132	0.428–2.992	
**Boarrmann Type**			< 0.001
I	Ref	Ref	
II	0.303	0.023–4.028	
III	0.187	0.021–1.668	
IV	0.923	0.110–7.733	
**T stage by CT scan**			0.049
T1	Ref	Ref	
T2	0.405	0.099–1.655	
T3	0.984	0.256–3.786	
T4a	3.088	0.577–16.528	
T4b	1.466	0.465–4.620	
**Serum CEA Level**	1.178	0.467–2.968	0.729
**Serum CA19-9 Level**	4.546	1.729–11.954	0.004
**N stage by CT scan**	4.240	2.299–7.818	< 0.001

Abbreviations: OR, odd ratio; CI, confidence interval; Ref: referent.

**Figure 1:**
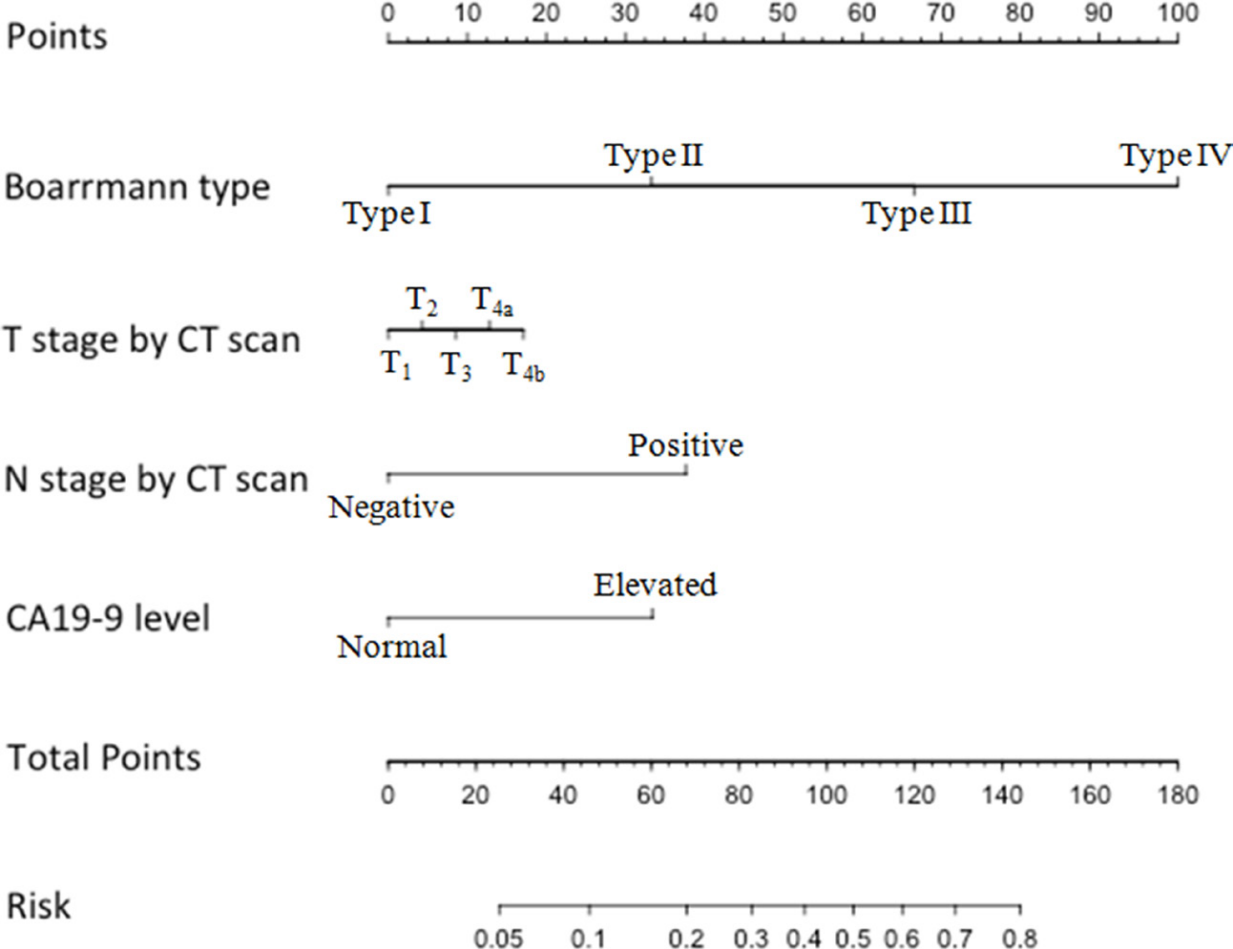
Nomogram for predicting the probability of lymph node metastasis in gastric cancer There are 7 rows in the nomogram. The behavioral variables are presented in rows 2 to 5, and the points for each variable correspond to the scale in row 1. The points of the seven variables are added to the total points presented on the scale in row 6, which corresponds to the risk predictor of lymph node metastasis in row 7.

**Figure 2:**
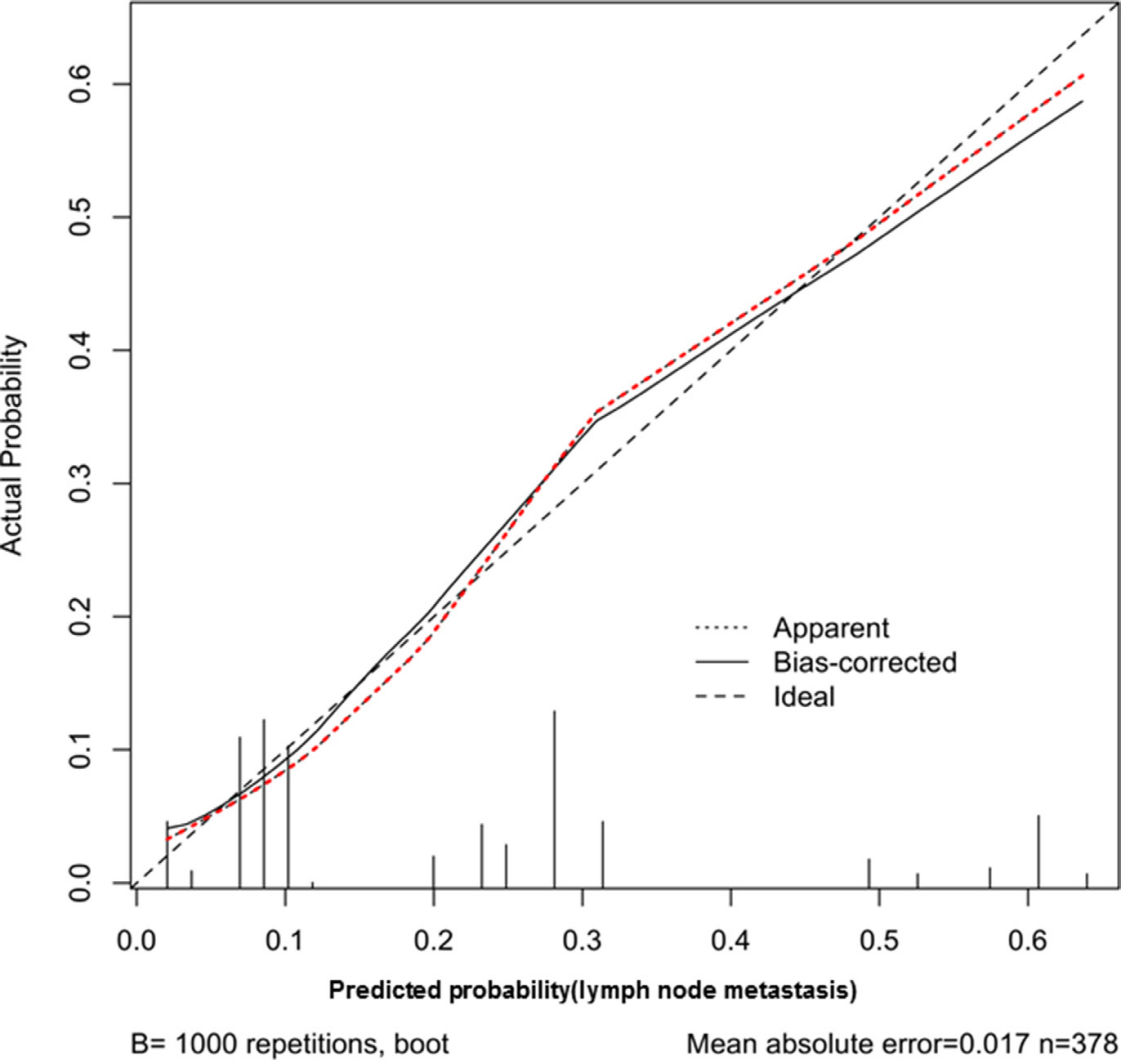
Calibration plot of the predictive model from the development cohort (*n* = 451): The actual probability versus the predicted probability The reference line represents perfect equality of the predicted probability and the actual incidence of lymph node metastasis.

Thus, we chose these four factors to develop a predictive nomogram for lymph node metastasis in gastric cancer patients. The nomogram corresponding to the model, including the possible factors that may increase the incidence of lymph node metastasis, is shown in Figure 1. The total score of each patient was calculated by the sum of the points determined by these four factors (Boarrmann type, T stage evaluated by CT, N stage evaluated by the CT and the preoperative serum CA19-9 level). Furthermore, we developed an internal calibration curve to validate the Nomogram model and found that the C-index was 0.786. (Shown in Figure 2).

### External validation of the nomogram model by the gastric cancer patients from the SYSUGIH

**Figure 3:**
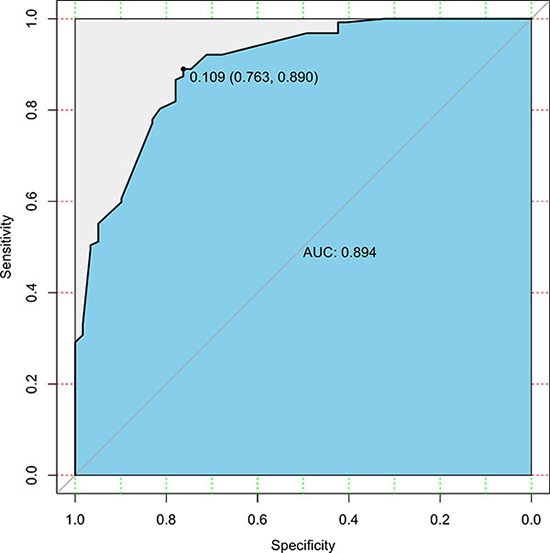
ROC curve of the predictive model for the validation cohort (*n* = 186) (ROC curve with an AUC value of 0.894; the cut-off value was set at 0.109) ROC, receiver-operating characteristic ROC; AUC, area under the ROC curve.

**Figure 4:**
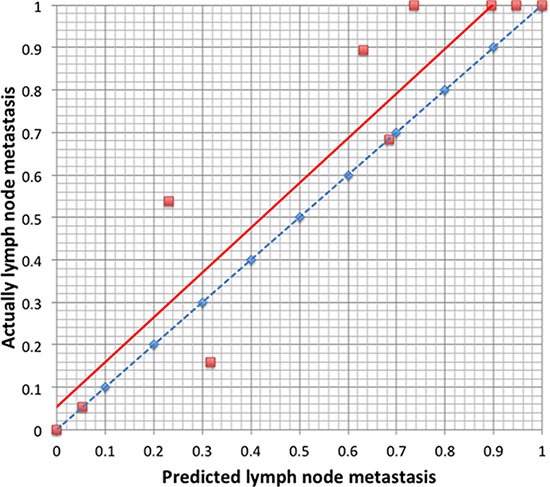
Calibration plot of the predictive model from the validation cohort (*n* = 186): The actual probability versus the predicted probability The model is sensitive in predicting lymph node metastasis with a low false-negative rate and a high false-positive rate.

We used the 186 gastric cancer patients from the SYSUGIH to estimate the predictive accuracy of the model. We developed an ROC curve for these patients. In this external validation, the C-index was 0.809, and the AUC was 0.894. The total accuracy of the prediction was 82.2%, and the false-negative rate was 5.4% with a cut-off value set at 0.109. The results are shown in Figures 3 and 4.

## DISCUSSION

Currently, clinicians often use the CT scan and the EUS to determine the N stage of the gastric cancer. However, the operative N stage of gastric cancer determined by CT scan and EUS were not satisfied. Previous studies showed that the accuracy of N stage by CT scan was 64–78% [13, 16, 17]; in addition, for EUS, the accuracy was also 50–71.2% [18–20]. It was still confusing for clinicians to identify the standard to determine whether the enlarged lymph node was a metastasis using CT or EUS. A length of more than 5 mm or 10 mm have both been used as the standard, but neither of these achieves the best effect in the preoperative staging of gastric cancer. PET-CT was also used in the preoperative judgement of lymph node metastasis and demonstrated its advantage on distant lymph node metastasis and bone metastasis [21]. However, the accuracy of PET-CT for regional lymph node metastasis did not demonstrate an advantage over CT or EUS [22]. In addition, it is not commonly accepted because of its radiation and expensive cost. Thus, we tried to obtain an accurate preoperative lymph node status by comprehensively considering the clinicopathological factors, including the tumor characteristics and its markers.

In our study, we used patients with a solitary lymph node metastasis and without lymph node metastasis to construct the model. We believed that patients with a solitary lymph node metastasis meant they were in the first or early step of lymph node metastasis. In our study, the cut-off value was set at 0.109, and under the validation, we found that the accuracy of the prediction of lymph node metastasis was 82.2%. In addition, the false-positive rate was 12.4%, and the false-negative rate was 5.4%. In the clinic, we know that approximately 10% of early stage gastric cancer patients will have lymph node metastasis, and we tried to ensure that they have the appropriate surgery with lymph node dissection but not ESD or EMR. In our study, the main purpose was to detect the existence of the lymph node metastasis to help surgeons make precise therapy decisions for the patients. A higher false-negative rate of the lymph node metastasis prediction and more EGC patients would have lymph node recurrence after ESD or EMR. Thus, we can accept the 0.109 as the cut-off value to reduce the false-negative rate of the prediction.

Previous studies have commonly proposed submucosal invasion as a significant risk factor predicting LNM [7, 15, 23, 24] and implied that T stage is the independent risk factor for lymph node metastasis. In the clinic, doctors always use CT scan and endoscopic ultrasound to evaluate the T stage of gastric cancer before surgery. Although CT can acquire a greater advantage in the evaluation of T stage of gastric cancer in the pathological T3 and T4 stages, with an accuracy of approximately 89% to 98%, it was still difficult to differentiate the T1 and T2 gastric cancer. In Luo's study, for example, the results showed that the evaluation accuracy of pathological stages T1 and T2 for gastric cancer by spiral CT was approximately 42.86%. In general, the accuracy rates in recent studies using multi-detector row CT for T and N staging have been reported to be 71.4–88.9% and 64–78%, respectively [22–25].

In our study, Boarrmann classification was also an important factor to predict lymph node metastasis before the surgery. We used the CT scan and endoscopy to observe the shape of the tumor to determine the preoperative Boarrmann classification of the patients. As we know, Boarrmann III and IV type gastric cancer always mean more invasiveness than the Boarrmann I and II type patients, and more invasiveness means an increased possibility of lymph node metastasis.

Actually, several nomograms were built to predict the lymph node status for gastric cancer. The purpose of our research was to select gastric cancer patients to undergo ESD or EMR precisely. In our study, we used a special group of gastric cancer patients who has a solitary lymph node metastasis or without lymph node metastasis to develop the nomogram model. We believe this model will have a high sensitivity for predicting lymph node metastasis in gastric cancer patients. As shown in our validation, the total accuracy of the validation group was 82.2%, with a false-negative rate of 5.4%. In addition, in our model, we only use 4 factors, which are easy to examine in the clinic to predict lymph node metastasis.

In 2014, The Cancer Genome Atlas (TCGA) network classified gastric cancer into the following four subtypes: Epstein-Barr virus (EBV)-positive tumors, microsatellite instable (MSI) tumors, gnomically stable (GS) tumors, and tumors with chromosomal instability (CIN) [23, 24]. A deep understanding of GC molecular characterizations has led to new therapeutic strategies, which may also help us to understand the molecular mechanism of lymph node metastasis in gastric cancer. We hypothesized that the gastric cancer patients with some special molecular characteristics might have the potential characteristics to promote lymph node metastasis. This molecular classification would help clinicians to predict lymph node metastasis based on the clinicopathological factors.

However, our study has several limitations. First, endoscopic ultrasound has been commonly used in the clinic to evaluate the stage of gastric cancer before surgery. However, our model group included patients from 2000 to 2012. In the early years, EUS was not widely used on these GC patients. Thus, we did not include the T and N stage evaluated by EUS in the nomogram model. Second, this study was a two centers, and the validation group has only 186 patients. We need more data to validate the model. Finally, we used the existence of lymph node metastasis as our primary goal, but the rate of lymph node metastasis of GC causes more and more concerns in recent research. How to balance the risk of LNM and the rate of LNM may be the next target. Finally, the Lauren classification was considered a risk factor for lymph node metastasis. However, there were too many deletions for the Lauren classification in both the development set and the validation set. Thus, the Lauren classification was not included in our research. Thus, our conclusions might need to be validated in the future.

We thought the AUC of the validation set was higher than the C-index from the development set because of the heterogeneity of the development set and the validation set. In our manuscript, we used a group of gastric cancer patients with a solitary lymph node metastasis or without lymph node metastasis. This nomogram model, developed by these groups of patients, is more sensitive to predict lymph node metastasis. However, in the validation group of patients, most of the patients had more than 1 lymph node metastasis, and the rate of the lymph node positive patients was higher than the development set. In addition, these two points may cause the AUC of the validation set to be higher than the C-index from the development set. In addition, we also need more data to validate this nomogram model.

In conclusion, this nomogram model can be used to predict lymph node status in gastric cancer patients. More data are needed to validate and optimize this model.

## MATERIALS AND METHODS

### Patients

The prospectively documented gastric cancer database was reviewed for patients who have undergone gastric cancer surgery with a curative intent between 2000 and 2012 at the Sun Yat-sen University Cancer Center (SYSUCC). The criteria for inclusion in this study were as follows:

(1). No synchronization malignant tumors;

(2). Radical D2 lymphadenectomy was performed, and more than 14 lymph nodes were harvested with a metastatic lymph node number of 0 or 1;

(3). Patient did not receive any neoadjuvant therapy, including chemotherapy, radiotherapy, and Chinese traditional medicine;

(4). No recurrence cases and gastric remnant cancer; and

(5). Complete follow-up data.

A total of 451 patients were included in our study as the development set. Among these, 350 patients (77.6%) were without lymph node metastasis, and 101 patients (22.4%) had solitary lymph node metastasis. The clinicopathological characteristics that were related to the lymph node metastasis are presented in Table 1.

The validation set consisted of patients with histologically proven gastric cancer who underwent a gastrectomy at Sun Yat-sen University Gastrointestinal Hospital (SYSUGIH) to verify the model. The criteria for inclusion in this study were as follows:

(1). No synchronization tumors;

(2). No neoadjuvant therapy, including chemotherapy, radiotherapy, and Chinese traditional medicine;

(3). No recurrence cases and gastric remnant cancer; and

(4). The pathological diagnosis of lymph node metastasis was complete.

A total of 186 patients were included in our study for the validation set. Among these, 59 patients (31.7%) were without lymph node metastasis, and 127 patients (68.3%) had lymph node metastasis.

### Follow-up

The follow-up included an outpatient follow-up, a telephone follow-up, a letter follow-up, a short message platform follow-up and an e-mail follow-up.

### Statistical analysis

All data were analyzed using the SPSS 20.0 statistical package (SPSS Inc., Chicago, IL, USA) and R version 2.11.1 (The R Foundation for Statistical Computing, Vienna, Austria). The associations between lymph node metastasis and the clinicopathological parameters were analyzed using the chi-square test (or Fisher's exact test when appropriate). Continuous variables were transformed into an adequate form to fit the proportional hazards and linearity assumptions. Risk factors for lymph node metastasis were studied using a binary logistic regression modeling technique.

A nomogram was developed as a tool for identifying patients at risk for lymph node metastasis, and it provided a graphical representation of the factors, which can be used to calculate the risk of lymph node metastasis for an individual patient by the points associated with each risk factor. The predictive accuracy of the model was graphically displayed using the receiver operating characteristic curve (ROC). The accuracy of the nomogram was then quantified using the area under the curve (AUC) for the validation. The threshold probabilities are arbitrary cutoff points used to classify patients as lymph node metastasis and non-lymph node metastasis. The sensitivity is defined as the probability of the model predicting a patient will have lymph node metastasis given that the patient has lymph node metastasis. The specificity is defined as the probability of the model predicting a patient will not have lymph node metastasis given that the patient does not have lymph node metastasis. Calibration was performed for the constructed nomogram, and the nomogram was internally validated using 1000 repetitions of bootstrap sample corrections and was externally validated by the database from the SYSUGIH. *P* values less than 0.05 were considered significant.
